# Comment on “Nonsyndromic Mandibular Symphysis Cleft”

**DOI:** 10.1155/2015/153787

**Published:** 2015-04-09

**Authors:** Keyur Mevada, A. Gopalakrishna

**Affiliations:** Global Hospital & Research Center, Mount Abu, Rajasthan 307501, India

The paper “Nonsyndromic Mandibular Symphysis Cleft” by Leela Krishna Guttikonda et al. was interesting. However, we would like to point out a factual error in the discussion in this paper. In the seventh paragraph of discussion the authors have attributed one theory for the formation of this cleft to Rana et al. for an article published in Indian Journal of Plastic Surgery in 2004. This is factually incorrect.

In that article the authors Rana et al. [1] have referred to an article by Oostrom et al. [2] in Plastic and Reconstructive Surgery in 1996 in which this hypothesis was propounded by the authors.

We feel that such errors should be avoided and due credit should be given to the first authors, namely, Oostrom et al.

## References

[1] Rana R. E., Puri V. A., Thakkur R. K., Blaiarsingh A. S. (2004). Median cleft of mandible and lower lip with ankyloglossia and ectopic minor salivary gland on tongue. *Indian Journal of Plastic Surgery*.

[2] Oostrom C. A. M., Vermeij-Keers C., Gilbert P. M., van der Meulen J. C. (1996). Median cleft of the lower lip and mandible: case reports, a new embryologic hypothesis, and subdivision. *Plastic and Reconstructive Surgery*.

